# Foot orthoses for adults with flexible pes planus: a systematic review

**DOI:** 10.1186/1757-1146-7-23

**Published:** 2014-04-05

**Authors:** Helen A Banwell, Shylie Mackintosh, Dominic Thewlis

**Affiliations:** 1 International Centre for Allied Health Evidence (iCAHE) School of Health Sciences, University of South Australia, Adelaide, SA 5001, Australia; 2 Biomechanics and Neuromotor Lab. School of Health Sciences, University of South Australia, Adelaide, SA 5001, Australia; 3 Sansom Institute for Health Research, University of South Australia, Adelaide, SA 5001, Australia

**Keywords:** Pes planus, Flat feet, Orthoses, Foot, Shoe inserts, Orthotic, Kinematics, Kinetics

## Abstract

**Background:**

Foot orthoses are widely used in the management of flexible pes planus, yet the evidence to support this intervention has not been clearly defined. This systematic review aimed to critically appraise the evidence for the use of foot orthoses for flexible pes planus in adults.

**Methods:**

Electronic databases (Medline, CINAHL, Cochrane, Web of science, SportDiscus, Embase) were systematically searched in June 2013 for randomised controlled, controlled clinical and repeated measure trials where participants had identified flexible pes planus using a validated and reliable measure of pes planus and the intervention was a rigid or semi-rigid orthoses with the comparison being a no-orthoses (shoes alone or flat non-posted insert) condition. Outcomes of interest were foot pain, rearfoot kinematics, foot kinetics and physical function.

**Results:**

Of the 2,211 articles identified by the searches, 13 studies met the inclusion criteria; two were randomised controlled trials, one was a controlled trial and 10 were repeated measure studies. Across the included studies, 59 relevant outcome measures were reported with 17 calculated as statistically significant large or medium effects observed with use of foot orthoses compared to the no orthoses condition (SMD range 1.13 to -4.11).

**Conclusions:**

No high level evidence supported the use of foot orthoses for flexible pes planus. There is good to moderate level evidence that foot orthoses improve physical function (medial-lateral sway in standing (level II) and energy cost during walking (level III)). There is low level evidence (level IV) that foot orthoses improve pain, reduce rearfoot eversion, alter loading and impact forces; and reduce rearfoot inversion and eversion moments in flexible pes planus. Well-designed randomised controlled trials that include appropriate sample sizes, clinical cohorts and involve a measure of symptom change are required to determine the efficacy of foot orthoses to manage adult flexible pes planus.

## Background

Pes planus (flat foot) is an umbrella term to describe feet with a visually lowered medial longitudinal arch often in association with rearfoot eversion [1,2]. Pes planus presents in two forms, described as rigid or flexible [3]. The World Health Organisation defines rigid pes planus as a congenital, rigid or spastic deformity of the foot and flexible pes planus as an acquired joint disorder resulting in a valgus foot deformity [4]. Rigid pes planus affects less than 1% of the population and leads to significant pain and disability often requiring surgical intervention [5,6]. Flexible pes planus reportedly affects between 2 to 23% of the U.S. adult population [1,7-9] with prognoses and intervention pathways remaining predominantly unclear, undefined and controversial [2,10,11]. Although well recognised within clinical practice and orthopaedic literature, no universally accepted classification and standardised measure of flexible pes planus exists [12-14]. Radiographic investigations are the reference standard to determine magnitude of pes planus; however, it is measured clinically using a variety of static foot posture indices, each with their own limitations [12,13,15].

Painful symptomatic presentations associated with flexible pes planus include: generalised lower limb pain; increased lower limb fatigue, Achilles tendinopathy, osteoarthritis, patellofemoral disorders and hip pain [1,16,17]. No agreement exists on the aetiology of flexible pes planus; however, frequently reported signs include abnormal rearfoot kinematics (e.g. excessive rearfoot eversion or increased range of rearfoot eversion), abnormal foot and ankle kinetics (e.g. elevated joint moments or abnormal loading forces) and altered physical function (e.g. altered muscle activation and timing or increased energy consumption) [18-21]. The symptoms of flexible pes planus have been attributed to the functional consequences of these signs [22] and intervention should be aimed at addressing these abnormalities [23].

Foot orthoses (FOs) are frequently prescribed interventions for flexible pes planus [24-26]. The most commonly prescribed FOs in Australia, the ‘modified Root device’ [27], were originally developed to influence the position of the subtalar joint towards a neutral position and reduce abnormal motion around this joint [28-30]. Subsequent views on the reported mechanism of how FOs affect the foot has evolved and, while variations exist, Kirby’s definition of “*an in-shoe medical device that alters magnitudes and temporal patterns of the reaction forces… and thus allowing for a more normal foot and lower extremity function that decreases pathological loading forces*” is often cited [31]. Therefore, based on these definitions, the use of FOs to alter the signs of flexible pes planus and ameliorate symptoms is plausible. The question is; does research evidence exist to support this notion? To our knowledge a systematic review investigating the use of FOs for a targeted adult flexible pes planus population has not been undertaken. With a limited understanding on how FOs impact on the signs and symptoms associated with flexible pes planus, there remains some controversy as to when and how clinicians determine if FOs are required [10,32-34]. Therefore, the aim of this systematic review was to assess the effectiveness of FOs to reduce foot pain, alter rearfoot kinematics and kinetics and improve physical function for adults with flexible pes planus.

## Methods

### Search strategy

**Figure 1:**
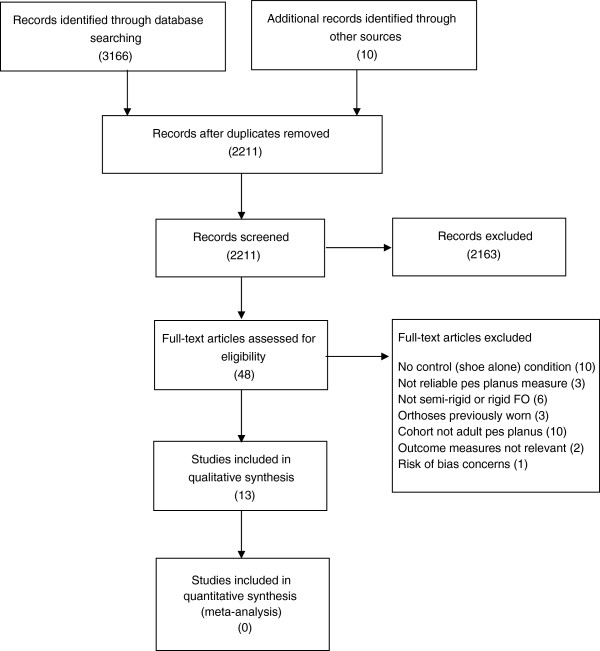
Flow chart of study selection.

The following databases were searched from inception to June 2013: Ovid Medline® (from January 1966 to date); CINAHL (from 1982 to date); Cochrane Central Register of Controlled Trials (CENTRAL) (issue 5, May 2013); Web of Science (from inception to date); SportDiscus (from inception to date); and Ovid Embase (1988 to date). Medical subject headings (MeSH) were exploded and combined with relevant keywords that were truncated where required. The search was limited to adult human subjects with no language restrictions applied. An example search strategy for Ovid Medline is outlined (Additional file 1). Further articles were sought from review of reference lists, conference proceedings and personal communications with content experts (Figure 1).

Two authors independently reviewed titles and abstracts of all studies generated by the search strategy using the inclusion and exclusion criteria. Consensus was reached for included studies through discussion with all three authors.

### Eligibility criteria

Studies were included if:

1. The study was published in a peer-reviewed journal.

2. The study design was a randomised controlled trial (RCT), controlled clinical trial (non-randomised intervention trial with a separate control group) or repeated measure intervention trial where participants acted as their own control.

3. Participants were adults with flexible pes planus defined using a reliable and validated foot morphology or posture index (median of available published reliability data of ICC ≥ 0.50) (Additional file 2).

4. The intervention was a rigid or semi-rigid FO where the orthoses were required to meet the standards defined by the Australian Podiatry Council for pre-formed or pre-moulded, moulded non-cast, moulded cast, or customised kinetic orthoses [35].

5. The comparison included a no-FO (shoes alone or flat non-posted insert) condition.

6. Outcome measures included a measure of foot pain/fatigue and/or functional changes in relation to: rearfoot kinematics (excessive rearfoot eversion, increased range or angular velocity of rearfoot eversion); foot kinetics (reduction of force, rearfoot joint moment or change in timing of force or moment) or physical function (changes in energy cost, lower limb muscle activation and timing, or postural control) during weight bearing, bipedal activities (standing, walking or running).

Studies were excluded if the participants had: a history of significant trauma, disease, inflammatory or systemic condition that may affect lower limb function (e.g. diabetes, neurological dysfunction, rheumatoid arthritis); had previously worn orthoses in the past six months; or the risk of bias of the study was deemed unacceptable (Figure 1).

### Risk of bias assessment

Risk of bias was assessed using the McMaster critical review form – Quantitative studies [36] and descriptive guidelines [37] which is applicable to RCTs, controlled clinical trials and repeated measure intervention trials. The tool has fifteen individual assessment points within eight domains. These are the study purpose, literature review, sample (described, justified, reliable), outcomes (reliable, valid), intervention (described, not contaminated, co-intervention/s avoided), results (statistically significant, appropriate analyses, clinically important), drop outs and conclusions (including clinical implications). The reporting of drop outs was not applicable for repeated measure trials and therefore was excluded from the critical review. Studies were awarded a ‘yes’ or ‘no’ rating for each individual assessment point of the remaining seven domains. A ‘yes’ rating was applied if the study completely fulfilled the criterion and a ‘no’ rating if the criterion was not completely fulfilled. Domains were required to have a ‘yes’ rating for a minimum of 50% of the individual assessment points to be considered acceptable. An *a priori* decision was set to include only studies deemed acceptable in four or more of the seven domains (Additional file 3). Risk of bias assessment was completed by two reviewers independently and discussions held until consensus.

The Australian National Health and Medical Research Council’s (NHMRC) evidence hierarchy was used to determine the level of evidence for each study with systematic reviews of RCTs considered high (level I) evidence, RCTs good (level II) evidence, pseudo-RCTs (controlled clinical trials) moderate (level III) evidence and repeated measure trials low (level IV) evidence [38].

### Data management

Data describing the sample characteristics, intervention characteristics, study protocol and adverse effects were extracted by two reviewers independently, from all studies, with consensus on results. Two reviewers independently extracted data with consensus on results. Novel measures, with no independent reliability or validity data, outcomes that were repetitious (within the same study) and those considered outside the scope of this review (extraneous) were excluded by consensus of three reviewers (Additional file 4). Means and standard deviations (SD) for each group were extracted where data were provided or supplied on request [34,39-44].

When more than one type or prescription approach of FOs were investigated, each device within the study was allocated simple identification (Device A, B etc.). Device A from one study is not comparable with Device A from an alternative study (Table 1).

**Table 1:** Table of included studies; level of evidence, cohort characteristics and type of FOs used

**Study**	**Level of evidence**	**Study design**	***N***	**Cohort**	**Mean age (SD)**	**Pes planus measure**	**Domain of outcome measures**	**FOs brand or type**	**FOs shell material**	**FOs posting**	
										**RF**	**FF**
Rome and Brown [48]	II	RCT	50 (25/25)	Excessive pronators	23.8 (2.2)	FPI-8	Physical function	Talar made ©	EVA	5°	0°
Esterman and Pilotto [34]	II	RCT	47 (25/22)	Air force recruits	21.6 (3.9)	AI	Pain	AOL®	Plastic	4°	4°
Otman et al. [50]	III-2	CCT	20 (20/20)	Female adults	25.8 (1.3)	Radiographs	Physical function	Arch supports	Polypropylene	0°	0°
Zifchock and Davis [41]	IV	RM	19	Low arched recreation runners	23.6 (6.4)	AHI	Rearfoot kinematics	A. Modified Root device	Graphite	0 – 7°	0°
								B. KLM®	Graphite	NR	NR
Mündermann et al. [47]	IV	RM	21	Recreational runners	25.4 (5.6)	RE	Rearfoot kinematics and kinetics	A. Modified Root device	Polypropylene	6 mm	6 mm
								B. Modified Root device	Polypropylene	0°	0°
								C. Pre-made Insert	EVA	6 mm	6 mm
Cobb et al. [52]	IV	RM	16	Low mobile arch adults	25.4 (6.5)	AHI	Rearfoot kinematics	A. MASS Device	Polyethylene composite	0°	0°
								B. Modified Root device	Leather and composite material	NR	NR
Murley et al. [40]	IV	RM	30	Adults	21.8 (4.3)	AI & NH	Physical function	A. Inverted (Blake) device	Polypropylene	20 °	0°
								B. Formthotic ®	Duel density polyethylene foam	6 mm	0°
Redmond et al. [44]	IV	RM	22	Excessive pronators	24	RE	Kinetics	A. Modified Root device	Polypropylene	6°	0°
								B. Pre-made Insert	Thin card with EVA posts	6°	0°
Zammit and Payne [42]	IV	RM	22	Excessive RF pronators (clinical practice)	44.3 (16.7)	FPI-8	Pain and physical function	Independently prescribed	Variable	IP	IP
Murley and Bird [40]	IV	RM	17	Adults	23.0 (5.0)	FPI-6	Physical function	A. Inverted (Blake) device	NR	30 °	0°
								B. Inverted (Blake) device	NR	15°	0°
								C. Inverted (Blake) device	NR	0°	0°
Hurd et al. [51]	IV	RM	15	Recreational runners	34.0 (10.0)	FF to RF	Rearfoot kinematics and kinetics	A. Flat foot Insert ©	Polyurethane with a poron layer	0°	4°
								B. SofSole®	Graphite Polyurethane	0°	0°
Johanson et al. [49]	IV	RM	22	Physical therapy attendees (clinical practice)	30.5 (8.0)	FF to RF	Rearfoot kinematics	A. Orthofeet Biothotics©	Water injected polyurethane shells with EVA posting	80% of FF post	Up to 7 mm
								B. Orthofeet Biothotics©	As above	80% of FF post	0°
								C. Orthofeet Biothotics©	As above	0°	Up to 7 mm
								D. Orthofeet Biothotics©	As above	0°	0°
Chen et al. [39]	IV	RM	11	Adults	45.9 (15.7)	AI	Kinetics and physical function	NR	Vinyl-acetate & 12% far-infrared nanopowders	NR	NR

RM – repeated measure. CCT – controlled clinical trial. AHI – arch height index, AI – arch index, FF to RF – forefoot to rearfoot relationship, NH – navicular height, FPI-8 – Foot Posture Index – 8- item version, FPI-6 – Foot Posture Index – 6- item version, RE- rearfoot eversion. MASS – maximum subtalar supination position, NR – not reported, FF – forefoot, EVA -ethyl vinyl acetate.

Orthotic Labs: Formthotic (Foot Science International, Christchurch, New Zealand) Flatfoot insert, (Hickory Brands, Inc, Hickory, NC, USA), Sofsole, LLC, Durham, NC), KLM (KLM Orthotic Laboratories; Valencia, CA, USA), AOL (Australian Orthotics Laboratory, International, Kirrance, NSW, Australia), Talar made (Talar Made Orthotics, Chesterfield, UK), Orthofeet Biothotics (Orthofeet Inc, Hillsdale, NJ, USA).

Outcomes are reported for pain, rearfoot kinematics, foot kinetics and physical function. All tabled data are displayed in descending level of evidence, followed by descending levels of assessed risk of bias concerns, followed by alphabetical order unless otherwise noted.

### Statistical methods

Sample size weighted standardised mean differences (SMD) and 95% confidence interval (CI) were calculated using Cochrane Review Manager (V.5) using the difference in mean scores between the FO and no-FO condition divided by the pooled standard deviation. Where the mean and standard deviations were not available for each condition, SMDs were calculated on mean difference divided by standard deviation of the mean difference, multiplied by the square root of two [45]. SMDs were considered statistically significant if the CI did not cross zero. Results are presented in forest plots where negative numbers favour the FO over the no-FO condition. Interpretations of the strength of the SMDs statistics were based on Cohen’s guidelines [46] with small effect ≥ 0.2, medium effect ≥ 0.5 and large effect ≥ 0.8. Statistically significant SMDs of less than 0.2 were considered very small.

## Results

### Study selection and design

Initial searches identified 2,211 studies. Two thousand, one hundred and sixty three (2,163) were excluded based on title and abstract. Forty eight studies were reviewed in full for eligibility, of which 35 studies were excluded based on inclusion and exclusion criteria (Figure 1) leaving 13 included studies (Table 1). No studies were high level of evidence, two studies were good level of evidence (RCTs), one study was moderate level of evidence (controlled clinical trial) and ten studies were low level evidence (repeated measure intervention trials).

### Risk of bias assessment

All studies had identified risk of bias (Additional file 3). While all included studies clearly stated their study purpose and the level of statistical significance, of the 13 included studies, only one justified the sample size [41]. Four studies had potential bias of co-interventions [41,42,47,48]. Individual studies had a risk of bias within the literature review [34], sample description [43], outcome measure reliability [39], outcome measure validity [49], intervention description [39,42,50], intervention contamination [39,51], appropriate analysis of results [39,50], clinical importance of results [49] and overall conclusion [34,39,49,51].

### Participants

In total, 312 participants were included in the 13 studies (Table 1). Most participants were young adults with 10 of the 13 included studies using cohorts aged 40 years or less. One study only recruited females [50]. Zammit and Payne [42] and Johanson et al. [49] recruited participants from clinical practice. Esterman and Pilotto [34] recruited air force cadets during basic training. Zifchock and Davis [38] and Mündermann et al. [44] both targeted recreational runners. Johanson et al. [46] and Hurd et al. [48] did not recruit from a specific population but required their participants to have forefoot varus of at least eight or five degrees respectively. The remaining studies did not report recruiting from specific cohorts. All studies recruited people with pes planus based on static foot posture. All studies involved 50 or less participants. Zifchock and Davis [41] had both high and low arch participants and did not report the low arch group separately. The authors kindly supplied data to isolate the low arch cohort.

### Types of foot orthoses

No two studies used the same FOs or approach to prescription (Table 1). Eight of the 13 studies involved two or more types of FOs with three of those comparing the same orthoses with different levels of customisation and/or posting (Table 1). Ten standardised the shell type and the level of rearfoot and forefoot posting across the cohort [34,39,40,43,44,47,48,50-52]. Two studies independently prescribed post levels, based on foot morphology, using different approaches [41,49]. One study investigated a combination of customised and prefabricated FOs individually prescribed [42] (Table 1).

### Outcome measures

**Figure 2:**
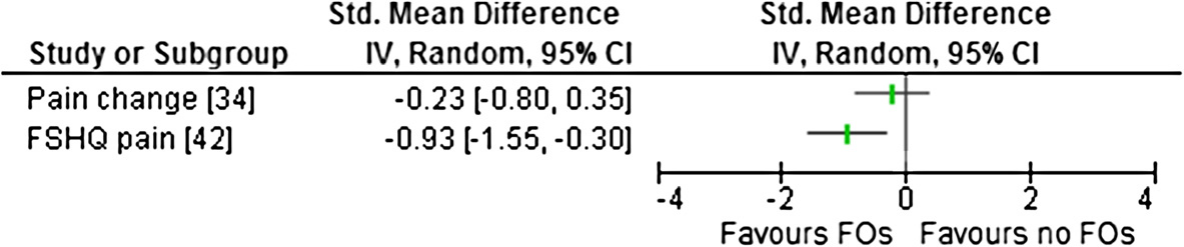
**Forest plot of data pooling for the use of FOs for the domain of pain.** FHSQ – foot health status questionnaire.

**Figure 3:**
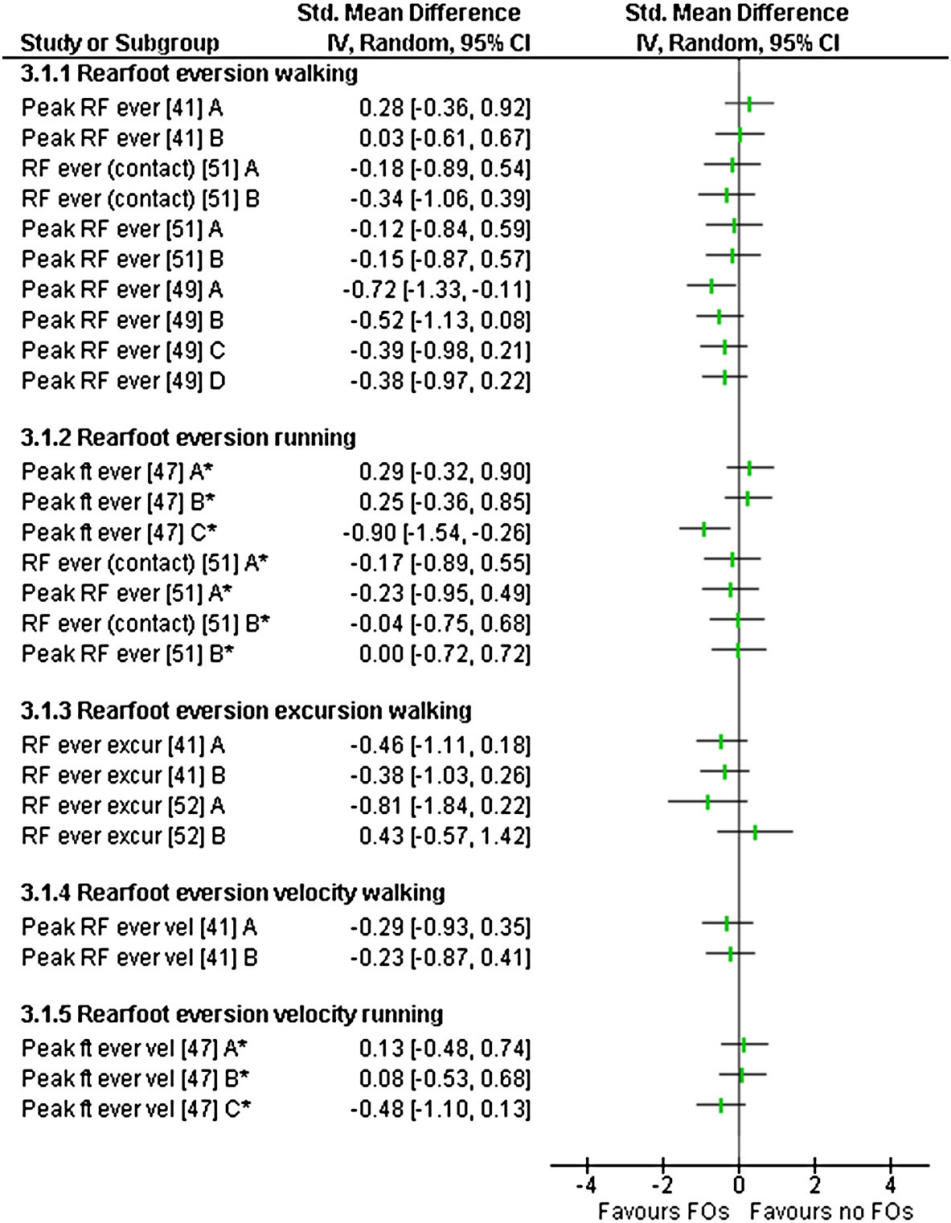
**Forest plot for data pooling for the domain of rearfoot kinematics.** RF – rearfoot, ever – eversion, ft – foot, excur – excursion, vel – velocity, *observed during running.

**Figure 4:**
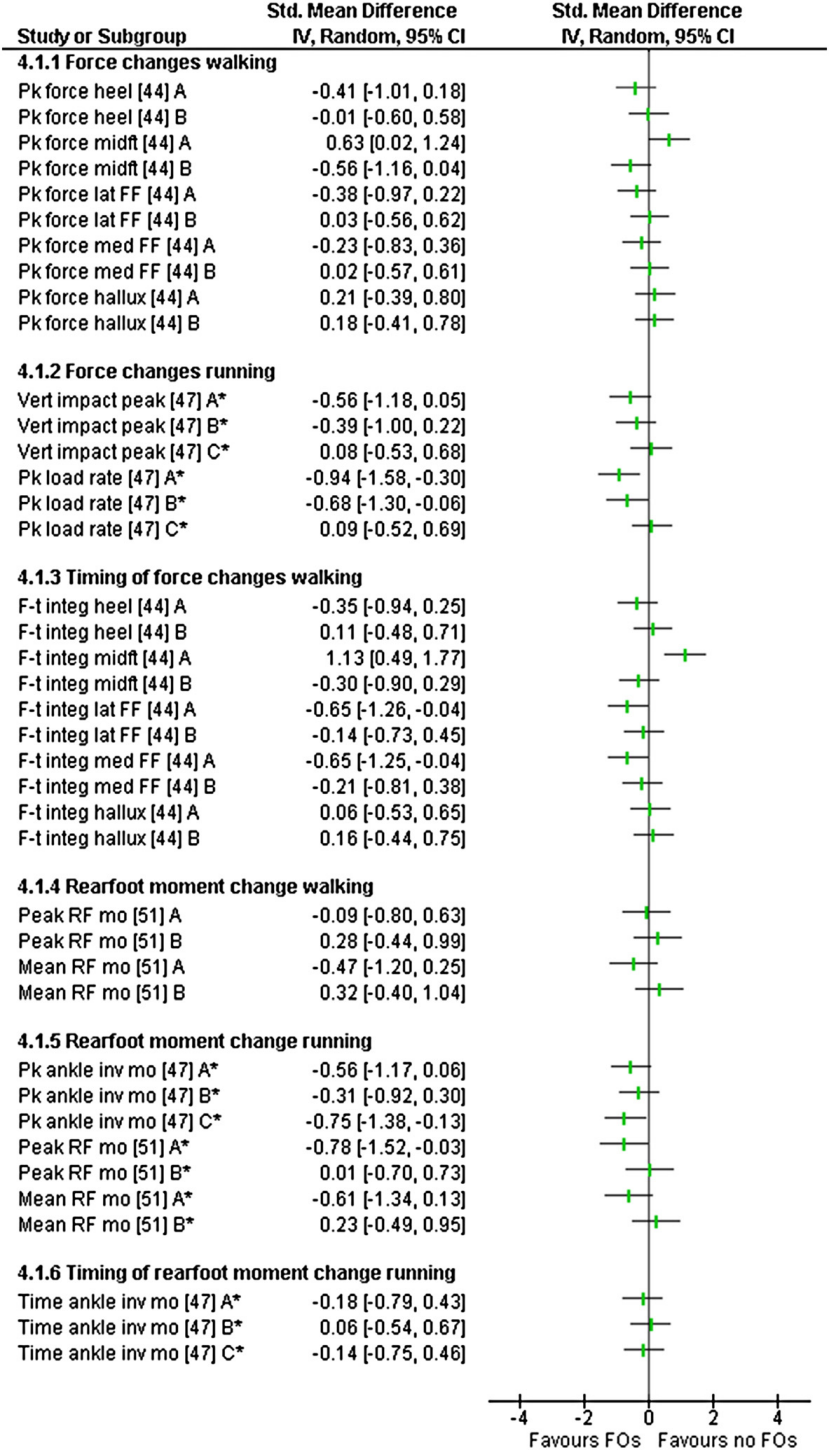
**Forest plot of data pooling for the domain of kinetics.** Pk – peak, FF – forefoot, lat – lateral, med – medial, vert – vertical, F-t integ – force time integral, RF – rearfoot, mo – moment, inv – inversion, *observed during running.

**Figure 5:**
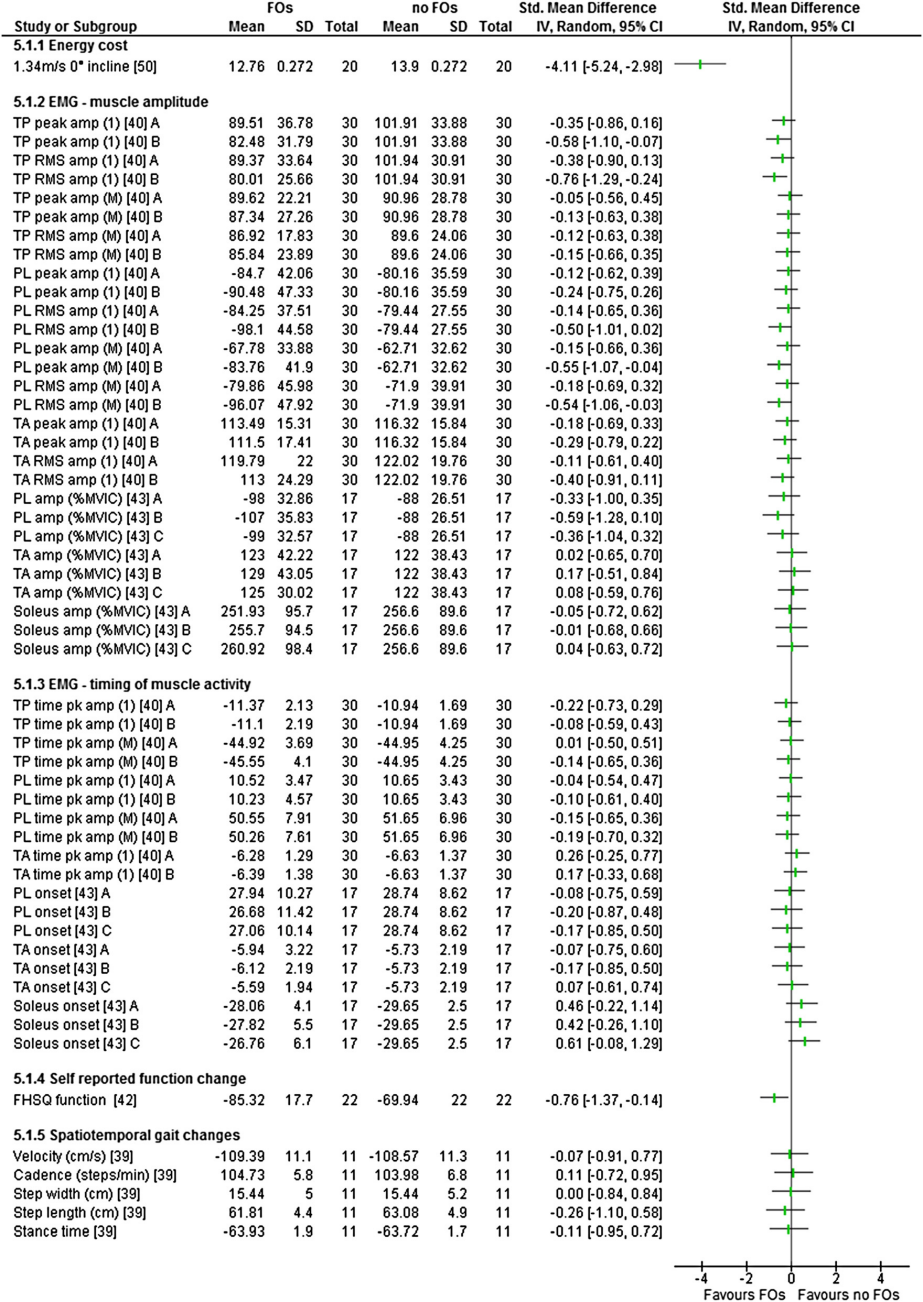
**Forest plot of data pooling for the domain of physical function.** EMG – electromyography, TP – tibialis posterior, RMS – root mean square, PL – peroneus longus, TA – tibialis anterior, MVIC – maximum voluntary isometric contraction, amp – amplitude, (1) – initial contact and loading, (M) – midstance, terminal and pre-swing.

All of the included studies used different outcome measures and measurement approaches (Tables 2, 3, 4 and 5). No study reported on adverse effects. The comparisons (SMD) between FO and no FO conditions for all relevant outcome measures are presented in Figures 2, 3, 4 and 5.

**Table 2:** Relevant outcome measures related to the domain of pain (significant SMD results are bolded)

**Study**	**Activity**	**Outcome**	**No FO (mean ± SD)**	**Device**	**FO (mean ± SD)**
Esterman and Pilotto [34]	10 week basic air force training recorded at baseline and 8 weeks	VAS 0 – 10, pain previous 24 hours following 8 weeks of FO use	1.14 (2.4)	NA	0.68 (1.5)
Zammit and Payne [42]	FHSQ reported at baseline and 4 weeks	Increase in FHSQ pain subscale following 4 weeks of FO use (reduction in pain)	49.84 (24.8)	NA	**70.86 (19.5)**

VAS – visual analogue scale, FO – foot orthoses, FHSQ – foot health status questionnaire, NA – not applicable.

**Table 3:** Relevant outcome measures related to the domain of rearfoot kinematics (significant SMD results are bolded) - walking unless otherwise noted

**Study**	**Activity**	**Outcome**	**No FO (mean ± SD)**	**Device**	**FO (mean ± SD)**
Zifchock and Davis [41]	Over-ground walking (2.0 m/s) in low arch cohort	Peak RF eversion (°)	4.31 (2.5)	A	5.45 (5.1)
				B	4.38 (2.2)
		RF eversion excursion (°)	10.60 (2.8)	A	9.47 (1.9)
				B	9.68 (1.8)
		Peak RF eversion velocity (°/s)	155.65 (46.9)	A	141.50 (47.2)
				B	144.78 (46.5)
Mündermann et al. [47]	Over-ground running (4.0 ± 0.2 m/s) in recreational runners with everted rearfoot.	Peak foot eversion (°) *	16.00 (2.3)	A	16.90 (3.6)
				B	16.60 (2.5)
				C	**13.70 (2.7)**
		Peak foot eversion velocity (°/s) *	464.70 (155.2)	A	484.40 (141.1)
				B	476.80 (145.0)
				C	392.90 (135.0)
Cobb et al. [52]	Over-ground walking (1.3 to 1.4 m/s) in low arch cohort	RF eversion excursion (terminal stance (°)	0.85 (0.8)	A	0.28 (0.5)
			0.02 (0.4)	B	0.18 (0.3)
Hurd et al. [51]	Over-ground walking (1.2 m/s ± 5%) in forefoot varus cohort	RF eversion (initial contact) (°)	-1.60 (3.6)	A	-1.00 (3.1)
				B	-0.30 (4.0)
		Peak RF eversion (loading) (°)	-3.40 (3.7)	A	-2.80 (5.6)
				B	-2.70 (5.3)
	Over-ground running (1.7 m/s ± 5%) in forefoot varus cohort	RF eversion (initial contact) (°) *	-3.30 (4.0)	A	-2.50 (5.0)
				B	-2.30 (4.5)
		Peak RF eversion (loading) (°) *	-5.60 (3.5)	A	-5.30 (10.5)
				B	-5.60 (11.1)
Johanson et al. [49]	Treadmill walking (1.11 m/s) in forefoot varus cohort	Peak RF eversion (°)	0.80 (3.0)	A	**-1.35 (2.8)**
				B	-0.88 (3.3)
				C	-0.44 (3.3)
				D	-0.36 (3.0)

FO – foot orthoses, RF – rearfoot, FF – forefoot, *during running.

**Table 4:** Relevant outcome measures related to the domain of kinetics (force and joint moment change) (significant SMD results are bolded) –during walking unless otherwise noted

**Study**	**Activity**	**Outcome**	**No FO (mean ± SD)**	**Device**	**FO (mean ± SD)**
Mündermann et al. [47]	Over-ground running (4.0 ± 0.2 m/s)	Vertical impact peak (N)	1499.10 (255.6)	A	1352.30 (233.6)
				B	1400.40 (242.5)
				C	1519.40 (265.9)
		Peak loading rate (N/s)	52.50 (11.1)	A	**42.0 (10.9)**
				B	**44.8 (11.1)**
				C	53.5 (11.9)
Redmond et al. [44]	Over-ground walking (self-selected speed)	Peak force (heel) (N)	544.50 (104.3)	A	501.90 (97.3)
				B	543.80 (100.6)
		Peak force (midfoot) (N)	195.30 (62.7)	A	**234.20 (58.8)**
				B	156.20 (73.9)
		Peak force (lateral FF) (N)	426.90 (12.1)	A	396.90 (110.3)
				B	429.90 (122.0)
		Peak force (medial FF) (N)	188.50 (62.9)	A	173.20 (65.4)
				B	190.00 (72.2)
		Peak force (hallux) (N)	148.90 (63.1)	A	161.40 (54.9)
				B	159.90 (53.9)
		Force-time integral (heel) (N/s)	1436.20 (462.2)	A	1285.00 (385.3)
				B	1488.90 (441.1)
		Force-time integral (midfoot) (N/s)	527.70 (224.4)	A	**812.30 (267.0)**
				B	454.40 (253.5)
		Force-time integral (lateral FF) (N/s)	1394.70 (575.1)	A	**1056.90 (436.3)**
				B	1317.00 (520.5)
		Force-time integral (medial FF) (N/s)	468.50 (212.1)	A	**340.30 (175.9)**
				B	423.50 (202.5)
		Force-time integral (hallux) (N/s)	294.90 (141.5)	A	304.60 (155.7)
				B	317.70 (145.3)
Mündermann et al. [47]	Over-ground running (4.0 ± 0.2 m/s)	Peak ankle inversion moment (Nm.kg^-1^)	0.47 (0.1)	A	0.40 (0.1)
				B	0.43 (0.1)
				C	**0.38 (0.1)**
		Time of ankle inversion moment (% of gait cycle)	41.00 (5.5)	A	39.90 (6.5)
				B	41.40 (6.7)
				C	40.20 (5.5)
Hurd et al. [51]	Over-ground walking (1.2 m/s ± 5%)	Peak RF moment (Nm.kg^-1^)	0.78 (0.4)	A	0.74 (0.5)
				B	0.88 (0.3)
		Mean RF moment (Nm.kg^-1^)	0.04 (0.4)	A	-0.18 (0.5)
				B	0.19 (0.5)
	Over-ground running (1.7 m/s ± 5%)	Peak RF moment (Nm.kg^-1^)	1.75 (0.7)	A	**1.23 (0.6)**
				B	1.76 (0.7)
		Mean RF moment (Nm.kg^-1^)	0.71 (1.0)	A	0.09 (1.0)
				B	0.92 (0.8)

FO – foot orthoses, DF – dorsiflexion, PF – plantarflexion, RF – rearfoot, NA – not applicable.

**Table 5:** Relevant outcome measures related to changes in: physical function (significant SMD results are bolded) – outcomes are measured during walking unless otherwise noted

**Study**	**Activity**	**Outcome**	**No FO (mean ± SD)**	**Device**	**FO (mean ± SD)**
Rome and Brown [48]	Quiet standing	Mean of the 300 balance points measured over 30 seconds (%)	49.40*^	NA	46.10*^
		Medial to lateral sway - rate of deviation from the mean balance over 30 seconds (%)	1.90*^	NA	1.30*^
		Anterior to posterior sway - rate of deviation from the mean balance over 30 seconds (%)	4.60*^	NA	4.80*^
Otman et al. [50]	Walking on treadmill at 1.34 m/s	Energy cost (ml/kg/min)	13.90*	NA	12.76*
Murley et al. [40]	Over-ground walking (self-selected ± 5%)	TP EMG peak amplitude (initial contact and loading)%	101.91 (33.9)	A	89.51 (36.8)
				B	**82.48 (31.8)**
		TP EMG RMS amplitude (initial contact and loading)%	101.94 (30.9)	A	89.37 (33.6)
				B	**80.01 (25.7)**
		TP EMG peak amplitude (midstance, terminal and pre-swing) (% of gait cycle)	90.96 (28.8)	A	89.62 (22.2)
				B	87.34 (27.3)
		TP EMG RMS amplitude (midstance, terminal and pre-swing)%	89.60 (24.1)	A	86.92 (17.83)
				B	85.84 (23.9)
		PL EMG peak amplitude (initial contact and loading)%	80.16 (35.6)	A	84.70 (42.1)
				B	90.48 (47.3)
		PL EMG RMS amplitude (initial contact and loading)%	79.44 (27.6)	A	84.25 (37.5)
				B	98.10 (44.6)
		PL EMG peak amplitude (midstance, terminal and pre-swing) (% of gait cycle)	62.71 (32.6)	A	67.78 (33.9)
				B	**83.76 (41.9)**
		PL EMG RMS amplitude (midstance, terminal and pre-swing)%	71.90 (39.9)	A	79.86 (46.0)
				B	**96.07 (47.9)**
		TA EMG peak amplitude (initial contact and loading)%	116.32 (15.8)	A	113.49 (15.3)
				B	111.50 (17.4)
		TA EMG RMS amplitude (initial contact and loading)%	122.02 (19.8)	A	119.79 (22.0)
				B	113.00 (22.3)
		TP EMG time of peak amplitude (initial contact and loading) (% of gait cycle)	10.94 (1.7)	A	11.37 (2.1)
				B	11.10 (2.2)
		TP EMG time of peak amplitude (midstance, terminal and pre-swing) (% of gait cycle)	44.95 (4.3)	A	44.92 (3.69)
				B	45.55 (4.1)
		PL EMG time of peak amplitude (initial contact and loading) (% of gait cycle)	10.65 (3.4)	A	10.52 (3.5)
				B	10.23 (4.6)
		PL EMG time of peak amplitude (midstance, terminal and pre-swing) (% of gait cycle)	51.65 (7.0)	A	50.55 (7.91)
				B	50.26 (7.61)
		TA EMG time of peak amplitude (initial contact and loading) (% of gait cycle)	6.63 (1.4)	A	6.28 (1.3)
				B	6.39 (1.4)
Zammit and Payne [42]	FHSQ reported at baseline and 4 weeks	Increase in FHSQ function subscale (increase in function)	64.94 (24.0)*	NA	85.32 (17.7)*
Murley and Bird [40]	Over-ground walking (self-selected speed)	PL EMG amplitude (% MVIC)	88.00 (26.5)	A	98.00 (32.9)
				B	107.00 (35.8)
				C	99.00 (32.6)
		TA EMG amplitude (% MVIC)	122.00 (38.4)	A	123.00 (42.2)
				B	129.00 (43.1)
				C	125.00 (30.0)
		Soleus EMG amplitude (% MVIC)	256.60 (89.6)	A	251.93 (95.7)
				B	255.70 (94.5)
				C	260.92 (98.4)
Chen et al. [39]	Over-ground walking (1.09 ± 0.11 m/s)	Velocity (cm/s)	108.57 (11.3)	NA	109.39 (11.1)
		Cadence (steps/min)	103.98 (6.8)	NA	104.73 (5.8)
		Step width(cm)	15.44 (5.2)	NA	15.44 (5.0)
		Step length(cm)	63.09 (4.9)	NA	61.81 (4.4)
		Stance time (%)	63.72 (1.7)	NA	63.93 (1.9)

FO – foot orthoses, EMG – electromyography, TP – tibialis posterior, PL – peroneus longus, TA – tibialis anterior, RMS – root mean square, MVIC – maximum voluntary isometric contraction, FHSQ – foot health status questionnaire, NA – not applicable, N/A – not available, *No FO condition was prior to orthoses dispense, the FO condition was following four weeks of orthoses use, ^ Median results and significance tabled as per original article– means and standard deviations unavailable.

### Pain

Two studies measured pain changes. One study (level II) reported no significant change in pain [34] and the other study (level IV) reported significant improvement in pain scores with FO use [42] (Table 2). Esterman and Pilotto [34] had a small, non-significant SMD for reducing ‘lower limb pain in previous 24 hours’ when comparing their intervention and control groups over eight weeks of basic training in air force recruits (Table 2). Data from Zammit and Payne [42] indicated a large SMD (mean difference of 21.02 points on the foot health status questionnaire (FHSQ) in reducing foot pain following four weeks of FOs use within a clinical cohort (Figure 2).

### Function change

#### *Rearfoot kinematics*

Five studies (level IV) analysed rearfoot kinematics during walking or running [41,47,49,51,52] with two studies reporting significant changes in kinematics with FOs use (Table 3).

Rearfoot eversion changes (peak and mean rearfoot eversion) were reported in four studies comparing eleven different types of FOs [41,47,49,51]. Significant decreases in rearfoot eversion were reported in two studies. Johanson et al. [49], noted peak rearfoot eversion was statistically significantly decreased (mean difference 2.15°) for device A during treadmill walking, with device B, C and D showing medium and small SMDs that were not statistically significant (Figure 3). Mündermann et al. [47] reported measures based on the foot modelled as a single rigid segment during overground running. Within this study, device C reduced foot eversion with a large SMD observed (mean difference 2.30°) in a cohort of recreational runners (Figure 3).

Two other studies investigated rearfoot eversion with non-significant results. Zifchock and Davis [41] during walking trials and Hurd et al. [51], during walking and running trials (Figure 3).

Rearfoot eversion excursions were reported in three studies [41,49,52] (Table 3). Zifchock and Davis [41] observed non-significant SMDs for both device A and B to reduce rearfoot eversion excursion (Figure 3). Cobb et al. [52] reported non-significant SMDs for rearfoot eversion excursion changes during terminal stance for both device A and B (Figure 3). Johanson et al. [49] reported no change to rearfoot excursion across all four FOs employed; however, data were not available to calculate SMDs.

Rearfoot eversion velocity was reported in two studies comparing five FOs (Table 3). Zifchock and Davis [41] observed non-significant SMD decreases during overground walking for device A and B (Figure 3). Mündermann et al. [47], during overground running, observed non-significant increases for device A and B, with a non-significant decrease observed with device C (Figure 3).

#### *Kinetics*

Kinetics of the foot were reported in four (level IV) studies [39,44,47,51] investigating change in loading forces and joint moments. Three of these studies reported at least one statistically significant change with FO use (Table 4).

Changes in force and its derivatives across the foot were reported in two studies [44,47]. Mündermann et al. [47] reported no significant change in vertical impact forces with all three devices (Figure 4). Peak loading rates were significantly altered for two of the three FOs used during overground running (Figure 4). A large SMD, which represented a reduction in loading rate, was observed with device A (mean difference 146.8 N/s). A medium SMD, which also represented a reduction, was observed with device B (mean difference 98.7 N/s). Device C had no effect (Figure 4). Mündermann et al. [47] also reported peak vertical ground reaction forces with similar results (Additional file 4). When using plantar pressure instrumentation, Redmond et al. [44] reported a significant, medium SMD, increase in peak force across the midfoot (mean difference 38.9 N) using device A (Figure 4). No significant results were reported across the heel, lateral and medial forefoot or hallux with device A during overground walking (Table 4). No significant changes across all foot regions were reported using device B (Table 4).

Force-time integrals from plantar pressure instrumentation were reported in one study comparing two FOs [44] (Table 4). Device A had a statistically significant large SMD effect in increasing the force-time integral across the midfoot (mean difference 284.6 N.s) and medium SMD reduction in the force-time integral at the forefoot (medial and lateral) (mean difference 128.2 and 337.7 N.s respectively)(Figure 4). Device B had no significant effects (Table 4). This study also reported mean force, peak pressures, mean peak pressures and pressure–time integrals (Additional file 4).

Changes in rearfoot joint moments were reported on in two studies [47,51] (Table 4). Mündermann et al. [47] reported reductions in peak ankle inversion moments during running for all three devices; however, only the medium SMD reduction effect observed for device C (mean difference 0.09 Nm.kg^-1^) was statistically significant (Figure 4). No significant differences were observed for the timing of the moment within the same study (Table 4). Hurd et al. [51], reported a significant reduction in peak rearfoot eversion moments during running with device A (mean difference 0.62 Nm.kg^-1^) of a large SMD; whereas device B had no significant effect (Figure 4). The same protocol, during walking, produced no significant differences using either FOs. Mean rearfoot eversion moments were altered with device A, with (non-significant) a small SMD reduction during walking (mean difference 0.53 Nm.kg^-1^) and (non-significant) a medium SMD reduction during running observed (mean difference 1.34 Nm.kg^-1^) (Figure 4). Conversely, using device B, small SMD increases were noted for both walking and running that were not statistically significant (Figure 4).

#### *Physical function*

Six studies reported on physical function changes. Outcome measures included postural sway [48], energy expenditure [50], electromyography (EMG) [40,43], FHSQ [42] and spatiotemporal gait variables [39]. One was level II [48], one was level III [50] and five were level IV [39,40,42,43]. Four of these studies reported significant changes in at least one outcome with FOs use (Table 5).

Postural sway in quiet standing was reported in Rome and Brown [48] in a RCT comparing FOs to shoes alone in two separate groups. Both groups were assessed in a shoes alone condition (session one) and again four weeks later (session two) where the intervention group were tested in FOs. A significant reduction in medial-lateral sway (32% improvement) was reported in the FOs group; however, data were not available to calculate SMDs (Table 5).

Energy expenditure changes were reported in a controlled clinical trial [50], with a large SMD observed within the pes planus group (Figure 5) indicating significant decreases in total energy cost during treadmill walking (mean difference 1.14 ml.kg.min^-1^) in comparison to the no-FO condition (Table 5). The non-pes planus (control) group remained consistent over the two testing sessions. This study also reported large energy expenditure savings during walking on inclines and positive effects for oxygen consumption for the pes planus group when using FOs during the same testing sessions (Additional file 4).

Changes in EMG of lower limb muscles during overground walking were reported in two studies, one measuring muscle activation and timing for two different FOs [40] and one measuring activation only for three different FOs [43] (Table 5). Murley et al. [40] reported a reduced tibialis posterior peak and root mean square (RMS) EMG amplitude at initial contact and loading (mean difference 19.4 and 21.9% respectively) with device B. Both these outcomes were calculated as a statistically significant medium SMD reduction in EMG amplitude (Figure 5). This was in conjunction with an increased peroneus longus peak and RMS EMG amplitude during midstance, terminal stance and pre-swing (mean difference 21.0 and 24.2% respectively) and RMS EMG amplitude during initial contact and loading (mean difference 18.6%) when using the same FOs (Table 5). These results were not reported with device A, nor were any other statistically significant SMDs reported using device A or B for tibialis anterior (Figure 5). This study also reviewed both devices influence on medial gastrocnemius (Additional file 4). In another EMG study [43], no statistically significant SMDs were observed for changes in amplitude of tibialis anterior, peroneus longus or soleus using three FOs with different posting levels (Figure 5).

Increases in self-reported function were reported (mean change 20.38 points on the FHSQ function subscale) (Table 5). A statistically significant medium SMD improvement was observed following four weeks of use of individually prescribed FOs in a cohort recruited from clinical practice [42] (Figure 5). Another study investigated gait velocity, step length and cadence and reported non-significant changes with FO use [39] (Table 5). This study also investigated stride length, reporting no effect (Additional file 4).

### Summary of results

From the 13 studies included, data were extracted for 59 relevant outcome measures related to the signs (rearfoot kinematics, foot kinetics and physical function) and symptoms (pain) associated with flexible pes planus (Tables 2, 3, 4 and 5). From these 59 outcome measures, 41 results reported across 13 studies were not statistically significant. Of the 13 studies reporting non-significant results, one was a level 11 [34] and 11 were level IV evidence [39-44,47-49,51,52]. Eighteen outcomes reported across eight studies were statistically significant (Table 6). Of the eight studies reporting statistically significant results, one was level 11 [48], one was level III [50] and six were level IV evidence [40,42,44,47,49,51]. Available data demonstrated that all statistically significant outcomes were a large or medium SMD effect when comparing the FO to the no FO condition (Table 6).

**Table 6:** Outcomes and device review categories of FOs where statistically significant SMDs were observed^ (in descending effect size) (during walking unless otherwise noted)

**Study**	**Level of evidence**	**SMD**	**95% CI**	**Domain**	**Specific outcome**	**Device**	**Difference in mean**
Otman et al. [50]	III	-4.11	-5.24 to -2.98	Physical function	↓ energy cost at 1.34 m/s, 0 incline (ml.kg.min-^1^)	NA	1.14
Redmond et al. [44]	IV	1.13	0.49 to 1.77	Kinetics	↑ force-time integral (midfoot) (N/s)	A	284.60
Mündermann et al. [47]	IV	-0.94*	-1.58 to -0.30	Kinetics	↓ peak loading rate (N/s)*	A	10.50
Zammit and Payne [42]	IV	-0.93	-1.55 to -0.30	Pain	↓ FHSQ pain subscale following 4 weeks of use (%)	NA	21.00
Mündermann et al. [47]	IV	-0.90*	-1.54 to -0.26	Rearfoot kinematics	↓ peak foot eversion (°) *	C	2.30
Hurd et al. [51]	IV	-0.78*	-1.52 to -0.03	Kinetics	↓ peak RF eversion moments (Nm.kg-^1^) *	A	0.52
Mündermann et al. [47]	IV	-0.75	-1.38 to -0.13	Kinetics	↓ peak ankle inversion moment (Nm)*	C	6.00
Murley et al. [40]	IV	-0.76	-1.29 to -0.24	Physical function	↓ TP EMG RMS amplitude (initial) (%)	B	21.93
Zammit and Payne [42]	IV	-0.76	-1.37 to -0.14	Physical function	↓ FHSQ function subscale following 4 weeks of use (%)	NA	20.40
Johanson et al. [49]	IV	-0.72	-1.33 to -0.11	Rearfoot kinematics	↓ peak RF eversion (°)	A	2.15
Mündermann et al. [47]	IV	-0.68	-1.30 to -0.06	Kinetics	↓ peak loading rate (N/s)*	B	7.70
Redmond et al. [44]	IV	-0.65	-1.26 to -0.04	Kinetics	↓ force-time integral (lateral FF) (N/s)	A	337.80
		-0.65	-1.25 to -0.04	Kinetics	↓ force-time integral (medial FF) (N/s)	A	128.20
		0.63	0.02 to 1.24	Kinetics	↑ force at midfoot (N)	A	38.90
Murley et al. [40]	IV	-0.58	-1.10 to -0.07	Physical function	↓ TP EMG peak amplitude (initial) (%)	B	19.43
Murley et al. [40]	IV	-0.55	-1.07 to -0.04	Physical function	↑ PL EMG peak amplitude (midstance +) (%)	B	21.05
Murley et al. [40]	IV	-0.54	-1.06 to -0.03	Physical function	↑ PL EMG RMS amplitude (midstance +) (%)	B	18.66

SMD values to the negative favour the FO condition over the no-FO condition.

FO – foot orthoses, RF – rearfoot, EMG – electromyography, TP – tibialis posterior, PL – peroneus longus, RMS – root mean square, FHSQ – foot health status questionnaire, NA – not applicable, *outcomes observed during running. ^Rome and Brown [48] SMDs unavailable.

The largest SMD (-4.11, CI -5.24 to -2.98) was observed in the domain of physical function (Table 6) in Otman et al.’s [50] controlled clinical trial that investigated changes in energy cost during treadmill walking, in a female pes planus group, with and without FOs (Table 6). These results favour the FO condition (Figure 5). The next largest SMD (1.13, CI 0.49 to 1.77) was within the domain of kinetics where Redmond et al. [44] investigated changes in the force-time integral at the midfoot (Table 6). This result favoured the no FO condition (Figure 4). This was the only statistically significant SMD effect that favoured the no FO condition (Table 6). Large effects within individual studies were also observed for reducing peak loading rates, pain and peak foot eversion (Table 6).

## Discussion

The aim of this systematic review was to assess the effectiveness of FOs to alter signs (abnormal rearfoot kinematics, abnormal kinetics and altered physical function) and symptoms (pain and fatigue) associated with flexible pes planus in adults. Key outcomes of this systematic review are that there are only two RCTs investigating the use of FOs for adults with flexible pes planus and that all studies had identified risk of bias issues (Additional file 3) and concerns regarding participant recruitment, choice of FOs or the outcome measures.

### Pes planus and participant recruitment

Each study used a different measure of pes planus and there was no consistency in recruitment criteria among studies. The majority of studies recruited cohorts of convenience based on static pes planus measures. It would seem more appropriate to investigate effectiveness of an intervention within a participant group who have recognised symptoms associated with the condition. One of the two studies that recruited from clinical practice reported large and medium SMD effects on pain and self-reported function [42] (Figures 2 and 5). These outcomes could, arguably, be the most clinically important measures included within this review.

### Approach to FOs

Concerns around the type of FOs employed were noted. The type of FOs selected for investigation in a study was rarely justified and often not described in detail. Ten of the included studies standardised the shell and approach to posting [34,39,40,43,44,47,48,50-52], only three studies individualised their approach [41,42,49]. A criticism of FOs intervention studies, anecdotally at least, is the justification of the FOs used and the apparent ad hoc approach to prescription options. This may be related to an absence of appropriate prescription guidelines to direct the researcher [53]. Currently there is no evidence to suggest that individually prescribing FOs offers any benefits over standardised devices, however if the FOs investigated in research are not mirroring those used in clinical practice then the evidence may also not be easily translated to clinical situations. The effect of the diversity of FOs used in the studies on the overall results of this review is unknown, and requires a further review with separate analyses. Further research into the impact of different approaches to the FOs used across all foot types is recommended along with the development of appropriate prescription guidelines to ensure future research outcomes are a genuine reflection of clinical practice results.

### Choice of outcome measures

The symptom of fatigue was not measured in any study and changes in pain levels were only investigated in two studies. This was surprising given that pain and fatigue are assumed to be common drivers for people with flexible pes planus to seek podiatric intervention [1,22,54]. Within this review, pain was significantly reduced when FOs were independently prescribed within a clinical cohort (Table 2); however, with no separate control group (or ‘sham device’ group) improvements in pain levels cannot be attributed to the FOs alone and may simply reflect pain resolving over time, a placebo or a Hawthorne effect [46].

Changes in rearfoot kinematics were frequently investigated (Table 3). Changes were noted predominantly in rearfoot eversion during walking with the measured reduction being significant (Figure 3). It is important to note that the actual magnitude of change is small (1.28 to 2.30°) and falls within accepted levels of measurement error [55]. It has also been suggested that this magnitude of effect on rearfoot kinematics is clinically meaningless and a direct link between rearfoot positioning and functioning has yet to be established [56]. Overall, the majority of rearfoot kinematic measures were not significant (Table 3).

Within the domain of kinetics, only two reported outcome measures demonstrated a statistically significant reduction for loading forces, both observed during running trials (Figure 4). Loading forces during walking were increased across the midfoot with the use of FOs and there was not a lateral shift of force demonstrated as expected (Figure 4). Results may have been influenced by both the use of an in shoe insole placed over the top of the orthoses and by the methods adopted for the quantitative analysis within this domain. The assumption was made that the goal of FO therapy was to reduce the overall force and decrease the time the force was applied [25]. Therefore, the increase in the amount and time of force measured across the midfoot was allocated as favouring the no FO condition (Figure 4). In essence, the actual clinical consequences of the changes in measured force and timing reported is not clearly understood [44] and interpretation of results should be viewed accordingly.

The impact on physical function is where the highest level of evidence was found (Table 5). Both medial-lateral sway during quiet standing and energy cost were positively affected with the use of FOs and offer good and moderate levels of evidence. This supports the historical belief that people with pes planus had reduced efficiency in gait, a belief that restricted entry into military service in the Australian, British and US armed forces for both World Wars [2]. Interestingly, while the use of FOs for improved stability in stance is supported within the literature [57,58], energy cost studies using FOs in other populations do not concur with the outcomes reported within this review. Hennacy [59] concluded that FOs, within a ‘foot problem’ group, induced an initial increase in energy consumption that returned to normal within three months. In a more recent study, Kelly [54] reported that no statistically significant changes were noted in energy cost during, and following, a one hour run with and without FOs in a non-pes planus cohort. Based on the outcomes of this systematic review, further investigations of energy cost and postural control within a flexible pes planus adult population is warranted.

### Limitations

Only a small number of studies were included in this review. All of the included studies had identified risk of bias with the assessment of risk of bias undertaken with a tool that allocated equal weight to all criteria (Additional file 3). Ten of the included studies are low level evidence (level IV) (Table 1) with all 10 being a repeated measure study design and eight of the 10 studies investigating two or more types of FOs. These studies met the required inclusion criteria; however, their inclusion may have affected the conclusions. To manage data levels and reduce errors from the same participants appearing repeatedly in analyses, several outcome measures were excluded on the basis they were considered repetitious or extraneous (Additional file 4), the effect of excluding these data on the results reported remain unknown.

## Conclusion

Within the limits of this systematic review it was determined that low level evidence exists that FOs positively impact on rearfoot kinematics, kinetics and physical function in adults with flexible pes planus. The small number of studies included and the risk of bias within the studies mean the clinical implications of the results of this review are not known. There is little evidence that FOs reduce pain and no evidence that FOs reduce fatigue.

Without high level evidence to support the purported effects of FOs podiatric clinicians are faced with the quandary of working within the bounds of evidence based practice, balanced against potentially conflicting clinical experience. Foot orthoses, specifically Root and modified Root devices, have been used for over 40 years with ‘arch inserts’ pre-dating them by over a century [18]. This review indicates that there are measurable consequences to FO use for the flexible pes planus in adults but these impacts are minimal. Quality research with clinically relevant outcomes, based on protocols that mirror clinical practice is required. This will allow future research to direct the development of robust and effective intervention pathways that offer the best possible outcome to the flexible pes planus adult population.

## Abbreviations

FOs: Foot orthoses; NR: Not recorded; N/A: Not available; NA: Not applicable; RCT: Randomised controlled trial; RM: Repeated measure; CCT: Controlled clinical trial; AI: Arch index; AHI: Arch height index; FF: Forefoot; RF: Rearfoot; NH: Navicular height; FPI-6: Foot posture index – 6- item version; FPI-8: Foot posture index – 8- item version; RE: Rearfoot eversion; MASS: Maximum subtalar supination position; FV: Forefoot varus; EVA: Ethyl vinyl acetate; SMD: Standardised mean difference; CI: Confidence interval; FHSQ: Foot health status questionnaire; DF: Dorsiflexion; PF: Plantarflexion; EMG: Electromyography; TP: Tibialis posterior; PL: Peroneus longus; TA: Tibialis anterior; RMS: Root mean square; MVIC: Maximum voluntary isometric contraction; IP: Independently prescribed; SE: Standard error; MG: Medial gastrocnemius; LoE: Level of evidence; Amp: Amplitude; Ever: Eversion; Ft: Foot; Excur: Excursion; Vel: Velocity; Pk: Peak; Lat: Lateral; Med: Medial; Vert: Vertical; f-t integ: Force time integral; mo: Moment; inv: Inversion.

## Competing interests

The authors declare that they have no competing interests.

## Authors’ contributions

The protocol for the review was written by HB. Risk of bias, data extraction and analysis was undertaken by HB, SM & DT. All authors contributed to and approved of the final manuscript.

## Authors’ information

HB was funded by an Australian Postgraduate Awards scholarship during the course of this study.

## Supplementary Material

Additional file 1 Search strategy example, Ovid MEDLINE. Click here for file

Additional file 2 Intra- and inter-rater reliability for commonly used measures of pes planus. Click here for file

Additional file 3 **Risk of bias of included studies (alphabetical order).** McMaster critical review tool – quantitative studies. Click here for file

Additional file 4 Excluded data based on novel, repetitious or extraneous outcome measures (results reported as significant are bolded). Click here for file
