# Supplementary material for: Foot orthoses for adults with flexible pes planus: a systematic review
Source: J Foot Ankle Res. 2014 Apr 5;7:23. doi: 10.1186/1757-1146-7-23 (PMC4108129; doi:10.1186/1757-1146-7-23)
Supplement: Additional file 1 — Search strategy example, Ovid MEDLINE. [file 1757-1146-7-23-S1.docx]

Additional file 1: Search strategy example, Ovid MEDLINE.

|  | exp Foot |
| --- | --- |
|  | exp Flatfoot |
|  | Pes planus |
|  | Planovalgus |
|  | exp pronat$ (pronation, pronating) |
|  | Arch insert |
|  | Arch insole |
|  | Shoe insert |
|  | Shoe insole |
|  | exp ortho$ (orthotic, orthoses, orthosis) |
|  | 1 or 2 or 3 or 4 or 5 |
|  | 6 or 7 or 8 or 9 or 10 |
|  | 11 and 12 |
|  | Limit 13 to human, adult |
