# Supplementary material for: Foot orthoses for adults with flexible pes planus: a systematic review
Source: J Foot Ankle Res. 2014 Apr 5;7:23. doi: 10.1186/1757-1146-7-23 (PMC4108129; doi:10.1186/1757-1146-7-23)
Supplement: Additional file 2 — Intra- and inter-rater reliability for commonly used measures of pes planus. [file 1757-1146-7-23-S2.docx]

Additional file 2: Intra- and inter-rater reliability for commonly used measures of pes planus.

| **Measure** | **Overall reliability*** | **Source/s** | **Median ICC** | **Range** |
| --- | --- | --- | --- | --- |
| Rearfoot eversion | Moderate | Diamond et al 1989[[1](#_ENREF_1)]; Smith-Oricchio & Harris 1990[[2](#_ENREF_2)] ; Sell et al 1994[[3](#_ENREF_3)]; Sobel et al 1999[[4](#_ENREF_4)]; Evans et al 2003[[5](#_ENREF_5)] | Intra 0.75  Inter 0.74 | 0.53 to 0.90  0.25 to 0.92 |
| Navicular height^ | Good | Sell et al 1994[[3](#_ENREF_3)]; Evans et al 2003[[5](#_ENREF_5)]; Menz and Munteanu 2005[[6](#_ENREF_6)]; McPoil and Cornwall 2006[[7](#_ENREF_7)] | Intra 0.95  Inter 0.76 | 0.84 to 0.98  0.64 to 0.96 |
| Forefoot to rearfoot | Moderate | Diamond et al 1989[[1](#_ENREF_1)]; Evans et al 2003[[5](#_ENREF_5)] | Intra 0.86  Inter 0.64 | 0.82 to 0.93  0.58 to 0.77 |
| Foot Posture Index – 8 item version | Moderate | Evans et al 2003[[5](#_ENREF_5)] | Intra 0.81  Inter 0.58 | N/A |
| Foot Posture Index – 6 item version | Moderate | Cornwall et al 2008[[8](#_ENREF_8)] | Intra 0.92  Inter 0.57 | N/A |
| Arch Height Index | Good | Williams and McClay 2000[[9](#_ENREF_9)]; Butler et al 2008[[10](#_ENREF_10)]; Xiong et al 2010[[11](#_ENREF_11)] | Intra 0.94  Inter 0.85 | 0.94 to 0.96  0.81 to 0.99 |
| Arch index | Good | Cavanagh and Rodgers 1987[[12](#_ENREF_12)]; Xiong et al 2010[[11](#_ENREF_11)] | Intra 0.96  Inter 0.94 | NR |

*based on inter-rater reliability where moderate ≥ 0.50 and good ≥ 0.80.NR – not reported. N/A - not available, ^navicular height truncated and non-truncated to foot length

References:

1. Diamond JE, Mueller MJ, Delitto A, Sinacore DR: **Reliability of a Diabetic Foot Evaluation.** *Phys ther* 1989, **69:**797-802.

2. Smith-Oricchio K, Harris B: **Interrater Reliability of Subtalar Neutral, Calcaneal Inversion and Eversion.** *J Orthop Sports Phys Ther* 1990, **12:**10-15.

3. Sell K, Verity T, Worrell T, Pease B, Wigglesworth J: **Two measurement techniques for assessing subtalar joint position: a reliability study.** *J Orthop Sports Phys Ther* 1994, **19:**162 - 167.

4. Sobel E, Levitz S, Caselli M, Brentnall Z, Tran M: **Natural history of the rearfoot angle: preliminary values in 150 children.** *Foot Ankle Int* 1999, **20:**119 - 125.

5. Evans A, Copper A, Scharfbillig R, Scutter S, Williams M: **Reliability of the Foot Posture Index and Traditional Measures of Foot Position.** *J Am Podiatr Med Assoc* 2003, **93:**203.

6. Menz H, Munteanu S: **Validity of 3 clinical techniques for the measurement of static foot posture in older people.** *J Orthop Sports Phys Ther* 2005, **35:**479 - 486.

7. McPoil TG, Cornwall MW: **Use of Plantar Contact Area to Predict Medial Longitudinal Arch Height During Walking.** *J Am Podiatr Med Assoc* 2006, **96:**489-494.

8. Cornwall M, McPoil T, Lebec M, Vicenzino B, Wilson J: **Reliability of the Modified Foot Posture Index.** *J Am Podiatr Med Assoc* 2008, **98:**7 - 13.

9. Williams D, McClay I: **Measurements used to characterize the foot and the medial longitudinal arch: reliability and validity.** *Phys Ther* 2000, **80:**864 - 871.

10. Butler RJ, Hillstrom H, Song J, Richards CJ, Davis IS: **Arch Height Index Measurement System: Establishment of Reliability and Normative Values.** *J Am Podiatr Med Assoc* 2008, **98:**102-106.

11. Xiong S, Goonetilleke RS, Witana CP, Weerasinghe TW, Au EYL: **Foot Arch Characterization: A Review, a New Metric, and a Comparison.** *J Am Podiatr Med Assoc* 2010, **100:**14-24.

12. Cavanagh P, Rodgers M: **The arch index: a useful measure from footprints.** *J Biomech* 1987, **20:**547 - 551.

13. Law M, Stewart C, Pollock N, Letts L, Bosch J, Westmorland M: **McMaster critical review form - Quantitative studies.** McMaster University Occupational Therapy Evidence-Based Practice Research Group.; 1998.
