# Supplementary material for: Foot orthoses for adults with flexible pes planus: a systematic review
Source: J Foot Ankle Res. 2014 Apr 5;7:23. doi: 10.1186/1757-1146-7-23 (PMC4108129; doi:10.1186/1757-1146-7-23)
Supplement: Additional file 3 — Risk of bias of included studies (alphabetical order). McMaster critical review tool – quantitative studies. [file 1757-1146-7-23-S3.docx]

Additional file 3: Risk of bias of included studies (alphabetical order). McMaster critical review tool – quantitative studies.

| **Author** | **Design** | **Study Purpose** | **Literature** | **Sample** | | **Outcomes** | | **Intervention** | | | **Results** | | | **Conclusion** |
| --- | --- | --- | --- | --- | --- | --- | --- | --- | --- | --- | --- | --- | --- | --- |
|  |  |  |  | Described | Justified | Reliable | Valid | Described | Not contaminated | Co-intervention avoided | Statistically significant | Analysis appropriate | Clinically important |  |
| Chen et al 2010 | Before & after | √ | √ | √ | x | x | √ | x | x | x | √ | x | √ | x |
| Cobb et al  2011 | cohort | √ | √ | √ | x | √ | √ | √ | √ | x | √ | √ | √ | √ |
| Esterman and Pilotto 2005 | RCT | √ | x | √ | x | √ | √ | √ | √ | x | √ | √ | √ | x |
| Hurd et al 2010 | Before & after | √ | √ | √ | x | √ | √ | √ | x | x | √ | √ | √ | x |
| Johanson et al 1994 | Before & after | √ | √ | √ | x | √ | x | √ | √ | x | √ | √ | x | x |
| Mündermann et al 2003 | Before & after | √ | √ | √ | x | √ | √ | √ | √ | √ | √ | √ | √ | √ |
| Murley and Bird 2006 | Before & after | √ | √ | x | x | √ | √ | √ | √ | x | √ | √ | √ | √ |
| Murley et al 2010 | Before & after | √ | √ | √ | x | √ | √ | √ | √ | x | √ | √ | √ | √ |
| Otman et al 1988 | cohort | √ | √ | √ | x | √ | √ | x | √ | x | √ | x | √ | √ |
| Redmond et al 2000 | Before & after | √ | √ | √ | x | √ | √ | √ | √ | x | √ | √ | √ | √ |
| Rome and Brown 2004 | RCT | √ | √ | √ | x | √ | √ | √ | √ | √ | √ | √ | √ | √ |
| Zammit and Payne 2007 | Before & after | √ | √ | √ | x | √ | √ | x | √ | √ | √ | √ | √ | √ |
| Zifchock and Davis 2008 | Cross-sectional | √ | √ | √ | √ | √ | √ | √ | √ | √ | √ | √ | √ | √ |

√ - yes, x – no. NA – not applicable, Studies were included if they were deemed acceptable in four of the seven domains. Domains were considered acceptable if ‘yes’ ratings were received for a minimum of 50% of the individual points.
