# Supplementary material for: Foot orthoses for adults with flexible pes planus: a systematic review
Source: J Foot Ankle Res. 2014 Apr 5;7:23. doi: 10.1186/1757-1146-7-23 (PMC4108129; doi:10.1186/1757-1146-7-23)
Supplement: Additional file 4 — Excluded data based on novel, repetitious or extraneous outcome measures (results reported as significant are bolded). [file 1757-1146-7-23-S4.docx]

Additional file 4: Excluded data based on novel, repetitious or extraneous outcome measures (results reported as significant are bolded).

| **Study** | **Level of evidence** | **Reasons for exclusion** | **Specific outcome** | **No FO (mean ± SD)** | **Device** | **FO (mean ± SD)** |
| --- | --- | --- | --- | --- | --- | --- |
| Otman et al [50] | III | Extraneous and repetitious | Energy cost (ml/kg/min) at 1.79 m/s, 10% incline | 21.07* | NA | **19.30*** |
|  |  |  | Energy cost (ml/kg/min) at 2.24 m/s, 20% incline | 30.79* | NA | **28.28*** |
|  |  |  | Oxygen consumption (ml.min^-1^) 1.34 m/s, 0% incline | 737.50 | NA | **677.50** |
|  |  |  | Oxygen consumption (ml.min^-1^) 1.79 m/s, 10% incline | 1115.50 | NA | **1023.00** |
|  |  |  | Oxygen consumption (ml.min^-1^) 2.24 m/s, 20% incline | 1618.50 | NA | **1493.50** |
| Mündermann et al [47] | IV | Repetitious | Peak foot inversion (°) | -0.30 (5.7) | A | **1.60 (6.2)** |
|  |  |  |  |  | B | **1.20 (5.9)** |
|  |  |  |  |  | C | -0.10 (5.6) |
|  |  |  | Peak foot inversion velocity (°/s) | 264.60 (97.7) | A | **291.30 (80.3)** |
|  |  |  |  |  | B | **293.80 (92.1)** |
|  |  |  |  |  | C | **236.70 (91.8)** |
|  |  |  | Time of peak ankle inversion moment (%) | 41.00 (5.5) | A | 39.90 (6.5) |
|  |  |  |  |  | B | 40.20 (5.5) |
|  |  |  |  |  | C | 41.40 (6.7) |
|  |  |  | Vertical active peak (N) | 1674.80 (205.6) | A | 1670.00 (201.6) |
|  |  |  |  |  | B | 1671.90 (204.1) |
|  |  |  |  |  | C | 1671.40 (208.3) |
| Cobb et al [52] | IV | Extraneous | RF complex DF displacement (midstance) (°) | 9.22 (2.2)^ | A & B | **10.10 (3.0)^** |
| Murley et al [40] | IV | Repetitious | MG time of peak amplitude (loading and pre-swing) (% of gait cycle) | 42.09 (3.1) | A | 42.80 (1.6) |
|  |  |  |  |  | B | 42.88 (3.2) |
|  |  |  | MG peak amplitude (loading and pre-swing) (% of gait cycle) | 104.78 (10.3) | A | 100.25 (11.7) |
|  |  |  |  |  | B | 103.54 (15.9) |
|  |  |  | MG RMS amplitude (loading and pre-swing) (% of gait cycle) | 103.66 (17.1) | A | **99.37 (15.7)** |
|  |  |  |  |  | B | 103.64 (24.4) |
| Redmond et al [44] | IV | Repetitious | Peak pressure (heel) (kPa) | 283.80 (56.8) | A | **220.60 (35.5)** |
|  |  |  |  |  | B | 278.60 (46.0) |
|  |  |  | Peak pressure (midfoot) | 220.20 (86.7) | A | **165.20 (43.3)** |
|  |  |  |  |  | B | **186.80 (50.1)** |
|  |  |  | Peak pressure (lateral FF) | 326.30 (101.7) | A | 322.50 (92.9) |
|  |  |  |  |  | B | 295.90 (68.0) |
|  |  |  | Peak pressure (medial FF) | 325.90 (86.5) | A | 320.40 (112.1) |
|  |  |  |  |  | B | 302.50 (91.4) |
|  |  |  | Peak pressure (hallux) | 268.40 (106.5) | A | **350.40 (122.4)** |
|  |  |  |  |  | B | 297.50 (79.6) |
|  |  |  | Mean peak pressure (heel)* | 140.00 | A | **119.20** |
|  |  |  |  |  | B | 140.30 |
|  |  |  | Mean peak pressure (midfoot)* | 80.30 | A | **60.50** |
|  |  |  |  |  | B | **63.90** |
|  |  |  | Mean peak pressure (lateral FF)* | 158.50 | A | **145.60** |
|  |  |  |  |  | B | 155.30 |
|  |  |  | Mean peak pressure (medial FF)* | 177.30 | A | 161.60 |
|  |  |  |  |  | B | 171.10 |
|  |  |  | Mean peak pressure (hallux)* | 140.30 | A | 152.80 |
|  |  |  |  |  | B | 148.40 |
|  |  |  | Pressure-time integral (heel) | 783.00 (248.8) | A | **640.00 (173.5)** |
|  |  |  |  |  | B | 796.00 (231.7) |
|  |  |  | Pressure-time integral (midfoot) | 710.90 (279.6) | A | 643.90 (196.3) |
|  |  |  |  |  | B | 641.70 (203.6) |
|  |  |  | Pressure-time integral (lateral FF) | 1107.70 (397.3) | A | **891.80 (318.7)** |
|  |  |  |  |  | B | **976.50 (304.9)** |
|  |  |  | Pressure-time integral (medial FF) | 840.10 (295.1) | A | **680.00 (289.3)** |
|  |  |  |  |  | B | 760.00 (260.4) |
|  |  |  | Pressure-time integral (hallux) | 752.90 (304.5) | A | 726.30 (384.60) |
|  |  |  |  |  | B | 684.70 (271.3) |
| Zammit and Payne [42] | IV | Novel outcome measures | Distance from reference point on shoe to lateral malleolus at forefoot contact (cm) | 5.26 (1.0) | NA | **5.48 (0.9)** |
|  |  |  | Distance from reference point on shoe to lateral malleolus at heel off (cm) | 5.48 (1.0) | NA | **5.72 (0.9)** |
|  |  |  | Distance from reference point on shoe to medial malleolus at forefoot contact (cm) | 3.58 (1.2) | NA | **3.32 (1.1)** |
|  |  |  | Distance from reference point on shoe to medial malleolus at heel off (cm) | 4.56 (1.3) | NA | 4.33 (1.3) |
| Murley and Bird [40] | IV | Repetitious | MG EMG amplitude (% MVIC) | 174.76 (12.2) | A | 176.40 (12.0) |
|  |  |  |  |  | B | 181.89 (11.9) |
|  |  |  |  |  | C | 174.76 (12.2) |
| Johanson et al [49] | IV | Repetitious | Peak calf to calcaneus (°) | 9.50 (2.8) | A | **7.25 (2.9)** |
|  |  |  |  |  | B | **7.65 (2.8)** |
|  |  |  |  |  | C | **8.00 (2.9)** |
|  |  |  |  |  | D | **8.22 (3.1)** |
| Chen et al [39] | IV | Extraneous and repetitious | Peak ankle DF moment (Nm.kg^-1^) | 0.12 (0.0) | NA | 0.13 (0.0) |
|  |  |  | Peak ankle PF moment (Nm.kg^-1^) | 1.38 (0.2) | NA | 1.22 (0.2) |
|  |  |  | stride length (cm) | 126.13 (5.9) | NA | 126.26 (7.9) |
|  |  |  | Peak ankle DF angle (°) | 23.58 (2.4) | NA | 24.04 (2.2) |
|  |  |  | Peak ankle PF angle (°) | 8.65 (2.3) | NA | 9.15 (3.1) |

FO – foot orthoses, DF- dorsiflexion, PF – plantarflexion, FF – forefoot, SE – standard error, MG – medial gastrocnemius, MVIC – maximum voluntary isometric contraction, RMS – root mean square NA – not applicable, *standard deviations unavailable from author, ^Article reported results for both FOs combined,
